# Influence of Adalimumab on the Expression Profile of Genes Associated with the Histaminergic System in the Skin Fibroblasts In Vitro

**DOI:** 10.1155/2018/1582173

**Published:** 2018-01-02

**Authors:** Dominika Wcisło-Dziadecka, Beniamin Grabarek, Nikola Zmarzły, Aleksandra Skubis, Bartosz Sikora, Celina Kruszniewska-Rajs, Joanna Gola, Urszula Mazurek, Eugeniusz Kucharz

**Affiliations:** ^1^Department of Skin Structural Studies, Chair of Cosmetology, School of Pharmacy with Division of Laboratory Medicine in Sosnowiec, Medical University of Silesia, ul. Kasztanowa 3, 41-200 Sosnowiec, Poland; ^2^Chair and Department of Molecular Biology, School of Pharmacy with Division of Laboratory Medicine in Sosnowiec, Silesian Medical University, ul. Jedności 8, 41-206 Sosnowiec, Poland; ^3^Department of Internal Medicine and Rheumatology, School of Medicine in Katowice, Medical University of Silesia, ul. Ziołowa 45/47, 40-635 Katowice, Poland

## Abstract

**Objective:**

The aim of this study was to evaluate the influence of adalimumab on expression profile of genes associated with the histaminergic system in Normal Human Dermal Fibroblast (NHDF) cells stimulated with 8.00 *μ*g/ml of adalimumab and the identification of miRNAs regulating these genes' expression.

**Methods:**

NHDFs were cultured with or without the presence of adalimumab for 2, 8, and 24 hours. The expression profile of genes and miRNA were determined with the use of microarray technology.

**Results:**

Among 22283 ID mRNA, 65 are associated with the histaminergic system. It can be observed that 15 mRNAs differentiate NHDFs cultures with adalimumab form control. The analysis of miRNAs showed that, among 1105 ID miRNA, 20 miRNAs are differentiating in cells treated with adalimumab for 2 hours, 9 miRNA after 8 hours, and only 3 miRNAs after 24 hours.

**Conclusion:**

It was also determined that miRNAs play certain role in the regulation of the expression of genes associated with the histaminergic system. The results of this study confirmed the possibility of using both genes associated with this system as well as miRNAs regulating their expression, as complementary molecular markers of sensitivity to the adalimumab treatment.

## 1. Introduction

Psoriasis is a chronic, immunological, inflammatory multifactorial skin disease which occurs in about 1–3% of the population [1–3] and coexists often with a metabolic syndrome [4–6]. Psoriasis vulgaris is the most common form of dermatitis, occurring in 90% of cases. Psoriasis has a negative impact on the quality of life and well-being of patients, because of visible dermatological changes and persistent skin itching [1].

This disease is associated with a high concentration of proinflammatory cytokines, such as interleukins (IL-12, IL-17, and IL-23), tumor necrosis factor *α* (TNF-*α*), interferon (IFN-*γ*), and transforming growth factor *β* (TGF*β*), in serum and skin lesions as a consequence of the immune system activation [7–10].

One of the most important cytokines in the induction and development of an inflammatory process is TNF-*α*, whose biological effects are mediated via two receptors, TNFR1 and TNFR2. In consequence, signaling pathways connected with this cytokine are activated. Increased secretion of TNF-*α* was observed in rheumatoid arthritis, ankylosing spondylitis, psoriasis, and inflammatory bowel disease [11–13].

In psoriasis, changes can be observed not only in mRNA expression, but also in miRNA profile. miRNAs (microRNAs) are a class of short and regulatory molecules of RNA that play an extremely important role in many human diseases. Currently, some miRNAs associated with skin and skin diseases including psoriasis have been identified and well described [14–17].

The high serum level of miR-1266 was observed in patients with psoriasis, highlighting its potential use as a biomarker [18]. In addition, the increased expression of miRNA-155, let-7i, miRNA-146a, miRNA-21, and miRNA-223 in the mononuclear cells of peripheral blood and also miRNA-21, miRNA-146a, and miRNA-223 in plasma was noted [19]. Lerman et al. observed the expression of the following miRNAs in psoriatic lesion skin: hsa-miR-149, hsa-miR-150, hsa-miR-210, hsa-miR-220, hsa-miR-326, has-miR-324-5p, hsa-miR-342, hsa-miR-326, hsa-miR-328, hsa-miR-345, hsa-miR-346, and hsamiR-197 [20].

One of the symptoms of psoriasis is skin itching, which can be associated with histamine secretion, mainly from immunocompetent cells, as a response to an inflammatory process. Histamine is a biogenic amine synthesized by decarboxylation of the amino acid L-histidine by histidine decarboxylase (HSD). Histamine is characterized as a mediator of a vast number of processes, such as inflammation, neurotransmission, physiological processes, smooth muscle contraction, and leukocyte chemotaxis. The biological effects of this amine are associated with its ability to interact with specific membrane receptors: H1, H2, H3, H4, and intracellular receptor Hic [21–23]. For example, the high concentration of histamine is observed in the respiratory system, skin, and gastric mucosal disorders. Only basophils and mast cells can store histamine in specific granules, from which it is released as a result of actions driven by various agents [11]. The histaminergic system not only consists of histamine and its receptors but also includes genes encoding proteins associated with transport and histamine metabolism, as well as genes encoding receptors participating in a signaling pathway activated by histamine.

Psoriasis is mainly treated by retinoids, cyclosporine A, and biological drugs. Biological medicines constitute a group of biologically active substances, typical proteins, and peptides, obtained with the application of molecular biology techniques and genetic engineering. The understanding of the pathomechanism inducing various diseases has created the possibility of developing drugs, the mechanism of action of which involves blocking the metabolic or signaling pathways that play a critical role in the disease development [24].

Adalimumab is a biological medicine, one of the three anti-TNF drugs used in the psoriasis vulgaris and psoriasis arthritis treatment [25]. This type of therapy is called a biological therapy, to be used in patients who have failed conventional treatment [26, 27]. This medicine is known as a fully human monoclonal antibody, which neutralizes tumor necrosis factor *α* (TNF-*α*). Adalimumab has an ability of binding to free and membrane-bound form of TNF-*α*, which prevents the interaction of TNF-*α* and receptors: TNFR1 and TNFR2; therefore, signaling pathways are blocked [28].

The aim of this study was to evaluate the influence of adalimumab on the expression profile of genes associated with the histaminergic system in Normal Human Dermal Fibroblast (NHDF) cells stimulated with 8.00 *μ*g/ml of adalimumab for 2, 8, and 24 hours and the identification of variously expressed genes whose transcriptional activity significantly differs.

## 2. Materials and Methods

### 2.1. Cell Culture

The NHDF cell line (fourth passage) was cultured in the FBM medium (Fibroblast Basal Medium; Lonza, Basel, Switzerland), supplemented with hFGF-B (Human Fibroblast Growth Factor-Basic) and insulin and gentamicin (FGM™ SingleQuots™; Lonza, Basel, Switzerland) at 37°C in a 5% CO_2_ incubator (Direct Heat CO_2_; Thermo Scientific, Waltham, MA, USA). The quantity of cells and their viability were monitored by cell counting in the Bürker chamber after staining them with 0.2% trypan blue (Biological Industries, Beit HaEmek, Israel). To test the effects of the drug on gene expression, 8 *μ*g/ml of adalimumab (AbbVie Biotechnology GmbH, Knollstrasse, Germany) was added to the cell culture for 2, 8, and 24 hours. Control cells were not treated with adalimumab. The concentration was customized to the level of adalimumab in the bodies of psoriatic patients.

### 2.2. Cytotoxicity Assay

An evaluation of the cytotoxicity of anti-TNF drug in NHDF cells was performed using the XTT assay (In Vitro Toxicology Assay Kit, XTT based; Sigma-Aldrich, St Louis, MO, USA).

The following range of adalimumab concentrations was used in the cytotoxicity test: 0.08 *μ*g/ml, 0.8 *μ*g/ml, 8 *μ*g/ml, 80 *μ*g/ml, and 800 *μ*g/ml, whereas the control sample involved the NHDF culture, which was not exposed to the drug. The following average absorbances were generated for the individual concentrations: 0.08 *μ*g/ml—0.079125; 0.8 *μ*g/ml—0.086625; 8 *μ*g/ml—0.08475; 80 *μ*g/ml—0.014375; 800 *μ*g/ml—0.023; control sample—0.087.

The statistical analysis of the XTT assay was performed with the use of Statistica 13.0 PL (StatSoft, Tulsa, Oklahoma, USA), assuming the statistical gravity factor *p* < 0.05. The statistical analysis involved Shapiro-Wilk data distribution normality tests, which was confirmed (*p* > 0.05) and then an ANOVA variance test was performed. Within the concentrations ranging from 0.08 *μ*g/ml to 8.00 *μ*g/ml no cytotoxic effect of the substance was observed on NHDF cells (*p* < 0.05), in turn, for adalimumab concentrations, 80 *μ*g/ml and 800 *μ*g/ml, a cytotoxic effect was observed when compared with the control sample; thus, the post hoc Tukey's test was conducted (80 *μ*g/ml versus C, *p* = 0.000141; 800 *μ*g/ml versus C, *p* = 0.000141).

### 2.3. Extraction of Total RNA

Total RNA was extracted from cells with the use of TRIzol reagent (Invitrogen Life Technologies, Carlsbad, CA, USA), according to the manufacturer's protocol. Extracts of total RNA were purified using the RNeasy Mini Kit (QIAGEN, Hilden, Germany) and treated with DNAse I (Fermentas International Inc., Burlington, ON, Canada), according to the manufacturer's instructions. The RNA concentration was determined with the use of a Gene Quant II spectrophotometer (Pharmacia LKB Biochrom Ltd., Cambridge, UK).

### 2.4. Oligonucleotide Microarray Analysis

The analysis of expression profile of genes associated with the histaminergic system was performed using commercially available oligonucleotide microarrays HG-U133A_2 (Affymetrix, Santa Clara, CA), according to the manufacturer's recommendations. Each gene chip contains 22238 probe sets that correspond to more than 18400 transcripts and 14500 well-characterized human genes.

About 8 *μ*g of total RNA was used for the cDNA synthesis using SuperScript Choice System (Gibco BRL Life Technologies). During the next step, cDNA was used as a template to produce biotin-labeled cRNA using BioArray HighYield RNA Transcript Labeling Kit (Enzo Life Sciences, NY). cRNA was then purified on RNeasy Mini Kit columns (Qiagen, Gmbh, Hilden, Germany). The biotin-labeled cRNA was fragmented using Sample Cleanup Module (Qiagen) and hybridized with the HG-U133A_2 microarray (Affymetrix). The cRNA hybridized to oligonucleotide arrays was stained with streptavidin-phycoerythrin conjugate and was scanned using GeneArray Scanner G2500A (Agilent Technologies, CA). The scanned data were processed for signal values using Microarray Suite 5.0 software (Affymetrix).

### 2.5. miRNA Microarray Analysis

The analysis of expression profile of miRNAs responsible for regulation of histamine-related genes was performed using commercially available GeneChip miRNA 2.0 Array (Affymetrix, Santa Clara, CA). The first step involved the labeling of miRNA with biotin by polyadenylation and ligation. The evaluation of labeling efficiency was verified using ELOS QC Assay (FlashTagBiotin HSR RNA Labeling Kit, Affymetrix). Subsequently, the hybridization of labelled RNA molecules of the microarray and probes was scanned using GeneArray Scanner 3000 7G (Agilent Technologies, CA). The scanned data were processed for signal values using Microarray Suite 5.0 software (Affymetrix).

### 2.6. Statistical Analysis

Agilent GeneSpring GX software was used for statistical analysis of the data after microarrays scanning. Differentially expressed genes were determined using one-way ANOVA (analysis of variance) test with asymptotic *p* values. The criteria used for differentially expressed genes required the absolute value of Fold Change (FC) to be greater than 1 (|FC| > 1) in at least one compared paired samples. The standard cut-off of *p* value < 0.05 was set to determine statistical significance of mRNA fluorescent signals.

The microarray data analysis was performed with the use of the GeneSpring 12.6.1 platform (Agilent Technologies, Inc., Santa Clara, CA, USA) and PL-Grid Infrastructure. In order to determine which of the differentiating miRNAs of the fibroblast cells exposed to the drug, compared to the control sample, could potentially affect the transcriptional activity of the differentiating mRNAs, the miRNA target prediction tools mirTAR (http://mirtar.mbc.nctu.edu.tw/human/predictionIndex.php) and microRNA.org (http://www.microrna.org/microrna/getGeneForm.do) have been used.

## 3. Results

### 3.1. Cytotoxicity Assay

The XTT assay revealed that no cytotoxicity was observed in NHDF cells after the adalimumab exposure for 2, 8, and 24 hours.

### 3.2. Transcriptome Analysis of NHDF Treated with Adalimumab

The names of the probes were obtained from Affymetrix NetAffxTM Analysis Center database (http://www.affymetrix.com/analysis/index.affx) on 20.09.2016. From 22 283 ID mRNA which are presented in the HG-U133A microarrays (Affymetrix), 65 are associated with the histaminergic system.

The first stage involved a one-way ANOVA testing with a Benjamini–Hochberg multiple testing correction, which showed 15 differentiating transcripts of histaminergic system genes (*p* < 0.05; Table 1).

Tukey's post hoc analysis test was used to identify differences in gene expression between the individual groups. In order to identify differentially expressed genes in cells treated with adalimumab, compared to the control group, the Tukey HSD post hoc test (*P* < 0.05 and FC *⩾* 1.0) was performed. It indicated 4 mRNAs with differentiable levels of expression between the control group (C) and the group consisted of cells exposed to adalimumab for 8 hours. Four mRNAs with differentiable levels of expression were identified between the control group (C) and the group of 24-hour samples. Additionally, 10 mRNAs with differentiable levels of expression were found between the control group (C) and the group of cells treated for 2 hours with adalimumab (Table 2).

**Figure 1:**
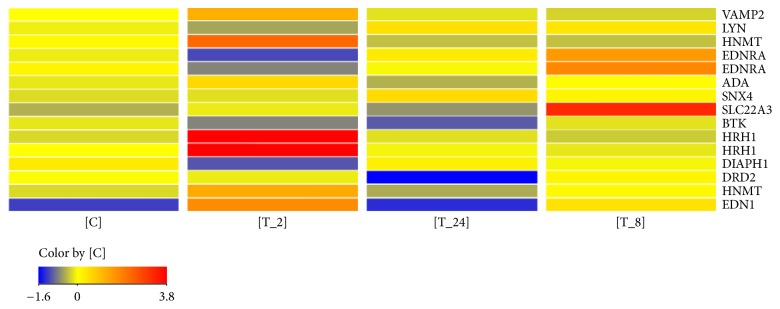
Heat map of fluorescence signal intensities of 15 mRNA IDs of genes associated with the histaminergic system (blue, the lowest value; red, the highest value of fluorescence signals; T_2, T_8, and T_24, study groups; C, control group).

A heat map was generated to assess the differences of the expression of 15 transcripts in individual groups (Figure 1).  Table 3 shows the changes.

**Figure 2:**
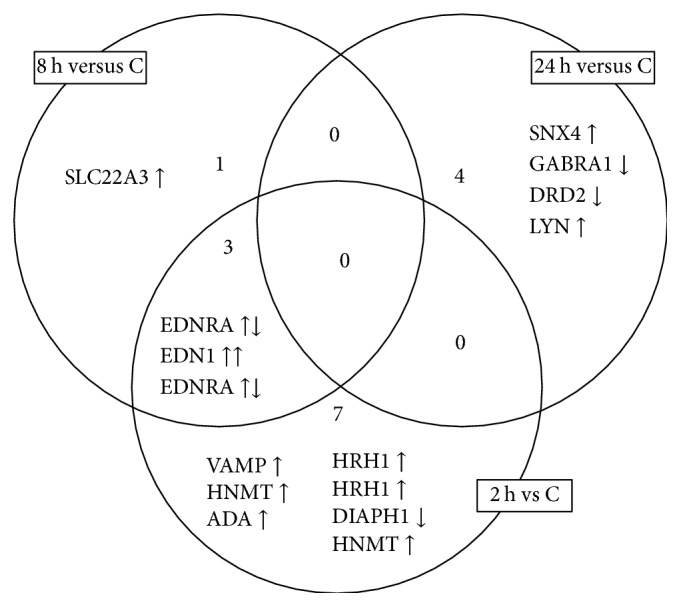
The Venn diagram of microarray results (*p* < 0.05).

A Venn diagram was drawn up to identify genes common to all the groups of transcriptomes and characteristic only for a particular group (Figure 2).

The analysis of Venn diagram showed that adalimumab changes the expression of 7 mRNAs encoded by 6 genes after 2 hours of stimulation of NHDF cells with the drug (VAMP2, HNMT, ADA, HRH1, DIAPH1, and HNMT), 4 mRNAs encoded by 3 genes after 8-hour stimulation (EDNRA, EDN1, and SLC22A3) with only one gene (SLC22A3) specific to cells treated with adalimumab for 8 hours. There were also changes in the expression of 4 mRNAs encoded by 4 genes after 24 hours of adalimumab stimulation (SNX4, GABRA1, DRD2, and LYN) (Table 4).

### 3.3. miRNA Microarray Analysis of NHDF Cells Treated with Adalimumab

The bioinformatic resources of the miRTar database have allowed to determine which of the differentiating microRNAs correlated to the mRNAs differentiating the fibroblasts exposed to the drug from the control group. Based on the miRNA SVR score parameter obtained from the microRNA database, the correlation between the respective miRNA molecule and the mRNA sequence of a gene was determined. The cut-off of −0.1 or lower is used as the cut-off point for this parameter based on the data from literature.

The microarray analysis in normal human skin fibroblast cells exposed for 2 hours with the anti-TNF drug revealed 20 miRNAs differentiating NHDF cells stimulated with adalimumab, compared to the control culture of dermal fibroblasts, but only 12 of them have connection with differentiating mRNAs.

An analysis of 8-hour exposure of fibroblasts to adalimumab revealed 9 differentiating miRNAs, but only one of them is connected with differentiating mRNA. We observed a connection between miR-1909 and gene encoding endothelin-1. The analysis showed increased expression of miRNA and EDN1.

**Figure 3:**
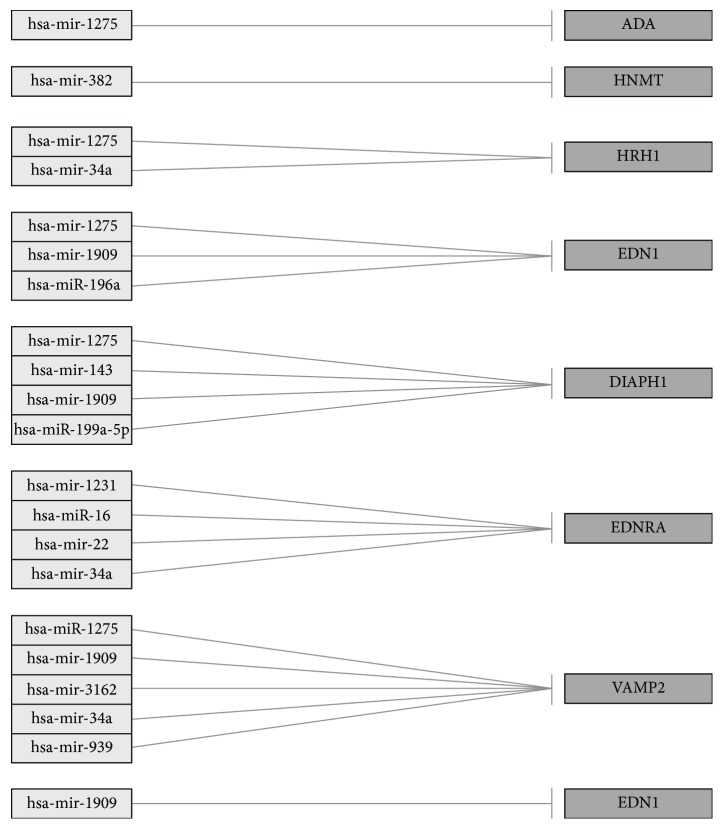
All miRNAs affecting the transcriptional activity of genes differentiating NHDF cells after 2 and 8 hours of stimulation with adalimumab from the control cells.

After 24 hours of the experiment, 3 miRNAs were identified as differentiating, but none of them showed any connection with differentiating mRNAs (Table 5 and Figure 3).

## 4. Discussion

Biological drugs are currently used to treat many diseases, such as psoriatic arthritis, psoriasis vulgaris, or Crohn's disease [29–32].

Adalimumab is a biological medicine, one of the three anti-TNF drugs used in the treatment of psoriasis vulgaris and psoriasis arthritis [25].

The results of described experiment show that the expression profile of genes associated with histamine activity is different in Normal Human Dermal Fibroblast (NHDF) cells treated with adalimumab and in the control cells. Moreover, it was observed that differences in expression depend on the drug exposure time. The highest amount of mRNA that differentiated normal human skin fibroblast exposed to anti-TNF drug was observed after 2 hours of drug stimulation. The number of differentiating transcripts decreased with the prolongation of the incubation time of skin fibroblasts with the drug. The ability to observe molecular changes that indicate cell sensitivity of skin fibroblasts after 2 hours following drug administration suggests the possibility of the application of genes associated with histaminergic system as complementary markers of the response to treatment.

In the authors' judgment, searching for potential markers that do not involve only blocking the signaling pathway of TNF by adalimumab will allow for greater precision and accuracy to monitor the effectiveness of therapy and to detect the lack of sensitivity of the cells to the drug at an early stage. In addition, it is important to remember that molecular changes precede phenotypic changes, which emphasizes the need to find new markers to change the treatment before the patient exhibits adverse changes in the phenotype.

As far as we are aware, this is the first study concerning both the regulation of transcriptional activity and changes in expression of genes associated with the histaminergic system in NHDH cells treated with adalimumab. We also assessed the miRNAs expression in the same conditions. Our aim was to find the relationship between genes of the histaminergic system and miRNAs that regulate these.

The most characteristic clinically observed symptom of psoriasis is parakeratosis (incomplete keratinization) caused by an abnormal proliferation and differentiation of keratinocytes in the basal layer of the skin [33–35].

The study of Gschwandtner et al. shows that histamine plays an important role in proinflammatory processes and deregulation of keratinocyte differentiation and impairs skin barrier in* in vitro* study model [36]. A higher concentration of this biogenic amine in psoriasis is associated with an activation and increased number of immune system cells, such as B lymphocytes, T lymphocytes, mast cells that can secrete proinflammatory cytokines, or other inflammatory compounds, for instance, TNF-*α*, interleukins, and also histamine [7–9]. The impact of histamine in inflammation was noted by Ruzicka and Glück who observed that after degranulation of mast cells, the tissue level of histamine can rise to 10–1000 *μ*M [37]; also the higher concentration of this compound was reported in patients with atopic dermatitis [38].

In this study, the following transcripts differentiating the NHDF cells treated with adalimumab in comparison to the control samples can be observed:* VAMP* (vesicle associated membrane protein 2),* HNMT* (histamine N-methyltransferase),* ADA* (adenosine deaminase),* HRH1* (histamine receptor H1),* DIAPH1* (diaphanous related formin 1),* HNMT* (histamine N-methyltransferase),* SLC22A3* (solute carrier family 22 member 3),* EDNRA* (endothelin receptor type A),* EDN1* (endothelin),* SNX* (sorting nexin 4),* GABRA1* (gamma-aminobutyric acid (GABA) A receptor, Alpha 1),* DRD2* (dopamine receptor D2), and* LYN* (LYN proto-oncogene, Src family tyrosine kinase). The highest number of differentiating mRNAs was observed after 2 hours of cells stimulation with drug, which may indicate the speed of drug action and the effect on genes expression.

This study focused on identifying the expression of genes associated with the histaminergic system in NHDF cells cultured in the presence of adalimumab (8 *μ*g/ml) for 2, 8, and 24 hours compared to control cells. After 2 hours of drug exposure, compared to the control sample, we observed changes in the expression pattern of the following genes:* VAMP2* (upregulated),* DIAPH1* (downregulated),* HNMT* (upregulated),* HRH1* (upregulated),* HRH1* (upregulated), and* ADA* (upregulated).

*VAMP2* was described as a gene encoding protein, associated with the neurotransmitter transport. Honardoost et al. analysed the role of* VAMP2* as one of the potential markers in an insulin resistance process. This hypothesis relied on the observation that* VAMP2* is essential in delivery of GLUT4 (glucose transporter type 4) to the plasma membrane. [39]. In this study, it can be observed that the change of* VAMP2* expression is statistically significant in NHDF cells after 2-hour exposure to adalimumab, in comparison to the control cultures.

*DIAPH1* is one of the three gene isoforms encoding mammalian diaphanous-related formins. Pan et al. indicate that* DIAPH1* is involved in megakaryocyte proplatelet formation, associated with remodelling either the actin or microtubule cytoskeletons [40]. Lin et al. observed the higher expression level of this gene in colorectal cancer and downregulation of transcriptional activity in colon carcinoma cells [41].

In this study, the effect of adalimumab on genes encoding proteins associated with metabolism of histamine,* HNMT (histamine N-methyltransferase)*, and histamine receptor H1,* HRH1*, can be observed.

The transcriptional activity of* HNMT* in NHDF cells after 2 hours of stimulation with adalimumab was higher than in nonstimulated cells. This gene encodes histamine N-methyltransferase involved in the biotransformation of histamine. Our study shows that adalimumab can influence the histaminergic system, reducing histamine concentration by increasing the expression of genes involved in its degradation.

The second gene directly associated with the histaminergic system is* HRH1*. Stimulation of this receptor by interacting with histamine leads to an activation of phospholipase C, inositol 1,4,5-triphosphate and diacylglycerol, protein kinase C, and Ca^2+^ [42].* HRH1* plays a fundamental role in the occurrence symptoms of immediate-type allergic reactions, activating nuclear factor *κ*B (NF*κ*B) and, in consequence, increasing the antigen-presenting capacity of the cells, expression of proinflammatory cytokines, and chemotactic factors [43]. The higher level of* HRH1* in cells treated with adalimumab, when compared to control cells, can be connected with an activation of immune mechanisms by the biological drug.

This study also shows that the higher mRNA concentration level of* ADA (adenosine deaminase)*, the gene encoding an enzyme involved in purine metabolism, catalysing hydrolysis of adenosine to inosine, can be observed after 2 hours of cells stimulation [44]. This enzyme plays a key role in the proliferation, maturation, and differentiation of lymphocytes and regulates adenosine concentration in blood plasma [45]. The higher level of* ADA* observed in this study may suggest the increased amounts of adenosine in NHDF cells after 2 hours of exposure to adalimumab as a result of ATP (adenosine triphosphate) degradation.

The statistical analysis shows that only* SLC22A3 (solute carrier family 22 member 3)* constitutes a statistically significant differentiating gene in the group of cells after 8 hours of exposure to adalimumab, in comparison to control cultures. It is believed that adalimumab can change transcriptional activity of* SLC22A3* (upregulated after 8 hours). This gene encodes transmembrane protein OCT3 (organic cation transporter 3), which plays an extremely important role in the outward transport of bioamines and some drugs, for example, metformin, histamine, and dopamine. [46–48].

The study of Li et al. have shown that deficiency of* SLC22A3* expression tends to increase the intracellular concentration of histamine [48].

Our data on expression profile of this gene may suggest that adalimumab decreases the level of histamine in cells after 2 and 8 hours of drug exposure; however, after 24 hours the amount of biogenic amines may be increased.

The study demonstrates that* EDNRA (endothelin receptor type A)* and* EDN1 (endothelin-1)* transcripts are differentiating after 2 and 8 hours of exposure to adalimumab, when compared to control. After 2 hours, both* EDN1* and* EDNRA* were overexpressed; on the other hand, after 8 hours the transcriptional activity of* EDNRA* was reduced.

Endothelin-1 is the most representative member of the endothelin family. It is known as a very strong vasoconstrictor factor, proliferation stimulator [49]. The interaction between endothelin-1 and endothelin receptor type A results in the activation of signaling pathways associated with apoptosis, angiogenesis, and cancerogenesis [50–53]. The expression pattern of* EDN1* and its receptor* EDNRA* may be explained by the anti-inflammatory properties shown by adalimumab. One of the functions of histamine is to increase the vascular permeability; thus, it can be observed after NHDF cells stimulation with adalimumab that genes associated with the opposite process to the one described, namely, the vasoconstrictor process, are overexpressed. The higher expression of these genes after 2 hours may also be caused by the secretion of endothelin from mast cell as a response to changes in the immunologic system after adalimumab administration.

Four genes,* DRD2*,* GABRA1*,* SNX4*, and* LYN*, are differentiating in NHDF cells treated with the drug, in comparison to control cultures. The changes in expression pattern of* DRD2 (dopamine receptor D2)* suggest the interaction between the histaminergic and dopaminergic systems and a complex effect of adalimumab on gene expression. In this study, the transcriptional activity of the gene in NHDF cells was downregulated after 24 hours of stimulation, when compared with control cell cultures.

After 24 hours of exposure to adalimumab, changes in the expression pattern of* GABRA1*, encoding gamma-aminobutyric acid type A receptor alpha 1 subunit, can be observed in NHDF cells. The results of the study show a decrease in the transcriptional activity of the said gene. Similar result was observed in brain samples taken from patients with multiple sclerosis [54]. The second differentiating gene after 24 hours was* SNX4 (sorting nexin 4)*, which, according to the Gene of NCBI (National Center for Biotechnology Information) database, encodes protein involved in the phosphoinositide transport.

It is demonstrated that the histaminergic system is complicated and not only consists of histamine and its receptors but also includes genes encoding proteins associated with the transport and metabolism of the amine and genes encoding receptors participating in signaling pathway activated by histamine. The results of changes in the transcriptional activity observed in this study indicate that genes associated with histaminergic system can be used as new markers of response to treatment with anti-TNF drug: adalimumab.

In this study, an attempt was made to select miRNAs regulating the transcriptional activity of genes associated with the histaminergic system in Normal Human Dermal Fibroblast (NHDF) cells exposed to adalimumab.

miRNA plays an important role in posttranscriptional regulation of gene expression, participating in many important physiological processes, including development, cell differentiation, and regulation of signaling pathways [55]. The participation of these molecules is emphasized in pathological conditions, for example, in tumors [55, 56], neurological disorders [56], and psoriasis [18].

miRNAs are a new group of biomarkers used to detect, predict, and monitor the effects of treatment in many diseases [57–60], which is determined by the high stability of these particles and the universality of their occurrence in body fluids, including blood, saliva, urine, and breast milk [61].

The study on the coexpression of miRNA and genes associated with the histaminergic system showed occurring several dependencies.

The ability of the miRNA to negatively regulate the transcriptional activity of a gene was determined by the miRNA SVR score. Based on the data for available literature, the cut-off value of this parameter is −0.1 or lower, because, for scores closer to zero, the probability of significant downregulation decreases, while the number of predictions increases rapidly [62].

The incubation of NHDF cell cultures for 2 hours with a biological drug leads to changes in the expression of 12 miRNAs (hsa-miR-1231, hsa-miR-1275, hsa-miR-143, hsa-miR-16, hsa-miR-1909, hsa-miR-196a, hsa-miR-199a-5p, hsa-miR-22, hsa-miR-3162, hsa-miR-34a, hsa-miR-382, and hsa-miR-939), regulating the expression of the analysed transcripts. The analysis also showed that a single miRNA may be involved in expression regulation of more than one mRNA transcript. This situation is observed during the 2-hour incubation of the cells with the drug for three miRNAs: hsa-miR-1275, which posttranscriptionally regulates the activity of following mRNAs:* ADA*,* HRH1*,* EDN1*,* DIAPH1*, and* VAMP2*; hsa-miR-34a:* EDNRA*,* VAMP2*, and* HRH1*; hsa-miR-1909:* VAMP2* and* DIAPH1*.

In our studies, we have observed that after 2-hour incubation of the cells with adalimumab, hsa-miR-22 is involved in the regulation of* EDNRA* expression. The data from the experiment show the overexpression of both hsa-miR-22 and* EDNRA* mRNA. Krintel et al. have demonstrated that miR-22 may be a useful diagnostic marker used to monitor the results of adalimumab therapy. Downregulation of mir-22 expression with simultaneous overexpression of miR-886.3p suggests the effectiveness of the treatment, while a reverse profile of these two miRNAs may be a negative prognostic marker in a pharmacotherapy [63].

It can be observed for the whole analysed group of 12 miRNAs that their overexpression is associated with increased expression of mRNA transcripts, regulated by the miRNA (except for identified decrease in the transcriptional activity of* DIAPH1* with the increased expression of regulating miRNAs: hsa-miR-1275, hsa-miR-143, hsa-miR-1909, and hsa-miR-199a-5p).

The analysis of cells treated for 8 hours with adalimumab shows an increase in the expression of miR-1909 and also* EDN1*, which is regulated by this miRNA.

The microarray analysis shows that there are only three differentiating miRNAs in cells treated for 24 hours with adalimumab. However, none of them is connected with any genes associated with histamine. It may suggest that the level of miRNA decreases with the exposure time.

miRNA-mediated regulation of gene expression in the anti-TNF therapy has been confirmed by Pivarsci et al. In his studies, he showed that anti-TNF therapy (etanercept) affects the change in the concentration of 38 miRNAs in the serum of patients with psoriasis treated with biological drugs [64]. In turn, Raaby et al. observed changes in the miRNA profile, during an adalimumab treatment in psoriatic skin biopsies.

The origin of miRNA in the RNA extract used in this study is unknown. An isolation of total RNA is associated with the presence of all types of RNA molecules in the extract. Therefore, it is possible that some of miRNA molecules in the generated extract originated from exosomes. Exosomes are membrane vesicles most likely secreted by cells of all types, involved in the intercellular communication. They contain a number of proteins, as well as the specific mRNAs and biologically active miRNAs regulating transcriptional activity of specific genes in target cells [65]. The exosomes may be responsible for the loss of efficacy of the analysed therapy. They are also used in the detection and evaluation of the severity of the condition [66].

The obtained results involving miRNAs indicate that the molecules play a certain role in the regulation of the expression of genes associated with the histaminergic system.

They also indicate that the expression profile of a particular gene can be controlled by several different miRNAs and it was observed that a single miRNA molecule acts as a regulator of more than one mRNA transcript.

In summary, based on the results of the described experiment, the anti-inflammatory effect of adalimumab has been confirmed, attributed to its ability to modulate expression of genes associated with the histaminergic system. The data also indicate the possibility to use the genes associated with the system and miRNAs regulating their expression as complementary therapeutic markers in personalized therapy. In addition, the sensitivity of cells to anti-TNF drug observed after two hours of skin fibroblasts incubation with the drug highlights the possibility to implement these molecular markers in routine diagnostics.

## Figures and Tables

**Table 1:** The results of the one-way ANOVA test with the Benjamini–Hochberg multiple testing correction.

Corrected *p* value	*p* all	*p* < 0.05	*p* < 0.02	*p* < 0.01	*p* < 0.005	*p* < 0.001
	65	15	11	10	7	5

**Table 2:** The Tukey HSD post hoc test results showing differentiating transcripts for cells treated with adalimumab in comparison to control cells.

Group name	Control	8 h	24 h	2 h
Control	15	4	4	10
8 h	11	15	8	11
24 h	13	10	15	14
2 h	5	4	1	15

**Table 3:** Transcripts of genes associated with the histaminergic system, differentiating the response of adalimumab treatment on NHDF cells, compared with the control samples.

ID	Gene symbol	2 hours (up/down)	FC (2 hours versus C)	24 hours (up/down)	FC (24 hours versus C)	8 hours (up/down)	FC (8 hours versus C)
201557_at	VAMP2	Up	1.3076547	Down	−1.037129	Down	−1.0554649
202625_at	LYN	Down	−1.0955737	Up	1.1316916	Up	1.1089189
204112_s_at	HNMT	Up	1.6368151	Down	−1.1016508	Down	−1.1010625
204463_s_at	EDNRA	Down	−1.226046	Up	1.09312	Up	1.4448898
204464_s_at	EDNRA	Down	−1.2038972	Down	−1.0421386	Up	1.4517964
204639_at	ADA	Up	1.1746593	Down	−1.0697097	Up	1.0407584
205329_s_at	SNX4	Up	1.0058819	Up	1.1812408	Up	1.0806441
205421_at	SLC22A3	Up	1.0740671	Down	−1.0358303	Up	2.274354
206678_at	GABRA1	Down	−1.1993337	Down	−1.282568	Down	−1.0323519
205579_at	HRH1	Up	2.4778538	Up	1.0098535	Down	−1.0161992
205580_s_at	HRH1	Up	2.3650975	Down	−1.0148736	Down	−1.0308013
209190_s_at	DIAPH1	Down	−1.3211001	Down	−1.0192791	Down	−1.082756
211624_s_at	DRD2	Down	−1.0191133	Down	−1.36553	up	1.0382545
211732_x_at	HNMT	Up	1.3924775	Down	−1.0599645	up	1.0668004
218995_s_at	EDN1	Up	1.8715541	Down	−1.0243433	up	1.3998785

**Table 4:** The changes of the mRNA expression in cells treated with adalimumab.

ID	Gene name	Encoded protein name	Group	Change
201557_at	VAMP2	Vesicle associated membrane protein 2	2 h versus C	Up
204112_s_at	HNMT	Histamine N-methyltransferase	2 h versus C	Up
204639_at	ADA	Adenosine deaminase	2 h versus C	Up
205579_at	HRH1	Histamine receptor H1	2 h versus C	Up
205580_s_at	HRH1	Histamine receptor H1	2 h versus C	Up
209190_s_at	DIAPH1	Diaphanous related formin 1	2 h versus C	Down
211732_x_at	HNMT	Histamine N-methyltransferase	2 h versus C	Up
205421_at	SLC22A3	Solute carrier family 22 member 3	8 h versus C	Up
204463_s_at	EDNRA	Endothelin receptor type A	8 h versus K i 2 h versus C	Up/down
204464_s_at	EDNRA	Endothelin receptor type A	8 h versus K i 2 h versus C	Up/down
218995_s_at	EDN1	Endothelin 1	8 h versus K i 2 h versus C	Up/up
205329_s_at	SNX4	Sorting nexin 4	24 h versus C	Up
206678_at	GABRA1	Gamma-aminobutyric acid (GABA) A receptor, alpha 1	24 h versus C	Down
211624_s_at	DRD2	Dopamine receptor D2	24 h versus C	Down
202625_at	LYN	LYN proto-oncogene	24 h versus C	Up

**Table 5:** The miRNA microarray analysis of NHDF cells treated with adalimumab versus control group.

Compared groups	ID	Name	FC	Change	Gene	SVR score
2 h versus C	hsa-miR-1231_st	hsa-mir-1231	2,24	Up	EDNRA	−0,2007

2 h versus C	hsa-miR-1275_st	hsa-mir-1275	−2,39	down	ADA	N/A
					HRH1	−0,5925
					EDN1	−0,8504, −0,6048
					DIAPH1	−0,0025
					VAMP2	−0,2280

2 h versus C	hsa-miR-143_st	hsa-mir-143	2,21	Up	DIAPH1	−0,1193

2 h versus C	hsa-miR-16_st	hsa-miR-16	2,17	Up	EDNRA	−0,0062, −0,0299

2 h versus C	hsa-miR-1909-star_st	hsa-mir-1909	2,86	Up	EDN1	−0,2846
					DIAPH1	−0,4198
					VAMP2	−0,2332

2 h versus C	hsa-miR-196a_st	hsa-miR-196a	3,13	Up	EDN1	−0,384

2 h versus C	hsa-miR-199a-5p_st	hsa-miR-199a-5p	3,49	Up	DIAPH1	−0,0020

2 h versus C	hsa-miR-22_st	hsa-mir-22	3,04	Up	EDNRA	−0,0720

2 h versus C	hsa-miR-3162_st	hsa-mir-3162	2,07	Up	EDNRA	−0,0102
					VAMP2	−0,7142

2 h versus C	hsa-miR-34a_st	hsa-mir-34a	6,52	Up	HRH1	−0,0024, −0,0017
					VAMP2	−0,7881

2 h versus C	hsa-miR-382_st	hsa-mir-382	2,82	Up	HNMT	−0,3818

2 h versus C	hsa-miR-939_st	hsa-mir-939	2,17	Up	VAMP2	−0,9181

8 h versus C	hsa-miR-1909-star_st	hsa-mir-1909	2,79	Up	EDN1	−0,2846
